# Elevated *PLA2G7* Gene Promoter Methylation as a Gender-Specific Marker of Aging Increases the Risk of Coronary Heart Disease in Females

**DOI:** 10.1371/journal.pone.0059752

**Published:** 2013-03-28

**Authors:** Danjie Jiang, Dawei Zheng, Lingyan Wang, Yi Huang, Haibo Liu, Leiting Xu, Qi Liao, Panpan Liu, Xinbao Shi, Zhaoyang Wang, Lebo Sun, Qingyun Zhou, Ni Li, Limin Xu, Yanping Le, Meng Ye, Guofeng Shao, Shiwei Duan

**Affiliations:** 1 Ningbo Medical Center Lihuili Hospital, Ningbo University, Ningbo, Zhejiang, China; 2 The Affiliated Hospital, Ningbo University, Ningbo, Zhejiang, China; 3 Zhejiang Provincial Key Laboratory of Pathophysiology, Ningbo University, Ningbo, Zhejiang, China; 4 Bank of Blood Products, Ningbo No.2 Hospital, Ningbo, Zhejiang, China; 5 Yinzhou People’s Hospital, Ningbo University, Ningbo, Zhejiang, China; VU University Medical Center, The Netherlands

## Abstract

*PLA2G7* gene product is a secreted enzyme whose activity is associated with coronary heart disease (CHD). The goal of our study is to investigate the contribution of *PLA2G7* promoter DNA methylation to the risk of CHD. Using the bisulphite pyrosequencing technology, *PLA2G7* methylation was measured among 36 CHD cases and 36 well-matched controls. Our results indicated that there was a significant association between *PLA2G7* methylation and CHD (adjusted P = 0.025). Significant gender-specific correlation was observed between age and *PLA2G7* methylation (males: adjusted r = −0.365, adjusted P = 0.037; females: adjusted r = 0.373, adjusted P = 0.035). A breakdown analysis by gender showed that *PLA2G7* methylation was significantly associated with CHD in females (adjusted P = 0.003) but not in males. A further two-way ANOVA analysis showed there was a significant interaction between gender and status of CHD for *PLA2G7* methylation (gender*CHD: P = 6.04E−7). Moreover, *PLA2G7* methylation is associated with the levels of total cholesterols (TC, r = 0.462, P = 0.009), triglyceride (TG, r = 0.414, P = 0.02) and Apolipoprotein B (ApoB, r = 0.396, P = 0.028) in females but not in males (adjusted P>0.4). Receiver operating characteristic (ROC) curves showed that *PLA2G7* methylation could predict the risk of CHD in females (area under curve (AUC) = 0.912, P = 2.40E−5). Our results suggest that *PLA2G7* methylation changes with aging in a gender-specific pattern. The correlation between *PLA2G7* methylation and CHD risk in females is independent of other parameters including age, smoking, diabetes and hypertension. *PLA2G7* methylation might exert its effects on the risk of CHD by regulating the levels of TC, TG, and ApoB in females. The gender disparities in the *PLA2G7* methylation may play a role in the molecular mechanisms underlying the pathophysiology of CHD.

## Introduction

DNA methylation often occurs in a CpG dinucleotide context and promoter DNA methylation can regulate the expression level of gene. Vertebrate CpG islands (CGIs) are short interspersed CpG-rich DNA sequences predominantly nonmethylated in or near approximately 40% of promoters of mammalian genes [1]. CGI hypermethylation of gene promoter usually silences gene expression [2]. Aberrant DNA methylation has extensively studied for the pathogenesis of multiple cancers including colorectal cancer [3], lung cancer [4], and leukemia [5]. However, only a few studies [6]–[10] have indicated an involvement of DNA promoter methylation in the susceptibility of coronary heart disease (CHD) that is the top killer in the world.

Gender disparities exist in the incidence, clinical presentation, diagnosis, and the surgical treatment of CHD [11]. For example, there are significantly more men die of CHD than women each year [11], [12]; Men suffered more from CHD and showed significantly more often chest pain localized on the right side of the chest [13]; Women were treated less intensively in the acute phase of acute coronary syndrome (ASC), while men were more often referred for coronary angiography [14]–[18]. Gender-related studies in CHD have identified a handful of biomarkers to clarify the differences between women and men [19]. It has been reported that by regulating expression of target gene, hormone-induced DNA methylation may increase or reduce the risk of complex diseases such as CHD [20]. Therefore, the gender difference in the epidemiological studies is likely attributable to the epigenetic modifications [21]–[23] such as DNA methylation.

*PLA2G7* gene product is a secreted enzyme whose activity is associated with CHD [24], [25]. Circulating lipoprotein-associated phospholipase A2 (Lp-PLA2) may indicate the inflammation level which plays a key role in the development of CHD [26], [27]. Lp-PLA2 is expressed abundantly in the necrotic core of coronary lesions [28], and once in the arterial wall facilitates hydrolysis of phospholipids [29]. *PLA2G7* gene expression and its expression quantitative locus were associated with CHD [30], [31]. In light of the previous findings, we hypothesized that promoter DNA methylation of *PLA2G7* gene in peripheral blood might contribute to the risk of CHD. Thus, the goal of this study was to assess whether *PLA2G7* gene promoter DNA methylation is associated with the risk of CHD and whether the association, if exists, is gender-specific.

## Materials and Methods

### Sample and Clinical Data

A total of 36 CHD cases and 36 age- and sex-matched controls were collected from the patients in the Ningbo Lihuili Hospital. The details of the inclusion criteria were presented in our previous publication [32]. All the individuals were Han Chinese originated from Ningbo city in the Eastern China. Blood samples were collected in 3.2% citrate sodium-treated tubes and then stored at −80°C. The study protocol was approved by the Ethical Committee of Ningbo Lihuili Hospital, and the informed written consent was obtained from all the subjects.

### Biochemical Analyses

Human genomic DNA was prepared from peripheral blood samples using the nucleic acid extraction analyzer (Lab-Aid 820, Xiamen City, China). DNA concentrations were determined by the ultramicro nucleic acid ultraviolet tester (NANODROP 1000, Wilmington, USA). Plasma levels of TG, TC, high density lipoprotein (HDL), and low density lipoprotein (LDL) were measured using an enzymatic end point assay [33]. The ApoA, ApoB and ApoE levels were measured by the transmission turbidimetric method [34]. The plasma Lp(a) concentrations were determined by a sandwich enzyme-linked immunosorbent assay method [Macra-Lp(a), SDI, Newark, Delaware]. The concentrations of ALT, AST, ALP and GGT in plasma were measured by the IFCC reference measurement systems [35]–[37]. The ALB level was worked through the Bromocresol green method [38]. All the tests applied the standard procedures recommended by the manufacturers. DNA methylation was measured by the pyrosequencing technology which combines sodium bisulfite DNA conversion chemistry (EpiTech Bisulfite Kits; Qiagen), polymerase chain reaction (PCR) amplification (Pyromark PCR Kit; Qiagen) and sequencing by synthesis assay (Pyromark Gold Q24 Reagents; Qiagen) of the CGI region on *PLA2G7* gene promoter. PCR primers were designed by PyroMark Assay Design software. Sequences of the PCR primers were shown in Table S1.

### Statistical Analyses

Using the SPSS package (version 16.0), a series of statistical analyses were performed to investigate the association of the promoter DNA methylation of *PLA2G7* gene with previous history of CHD and various biochemical factors. All the P values were adjusted for the history of smoking, diabetes and hypertension. A two-tailed p-value<0.05 was considered to be significant.

## Results

**Figure 1:**
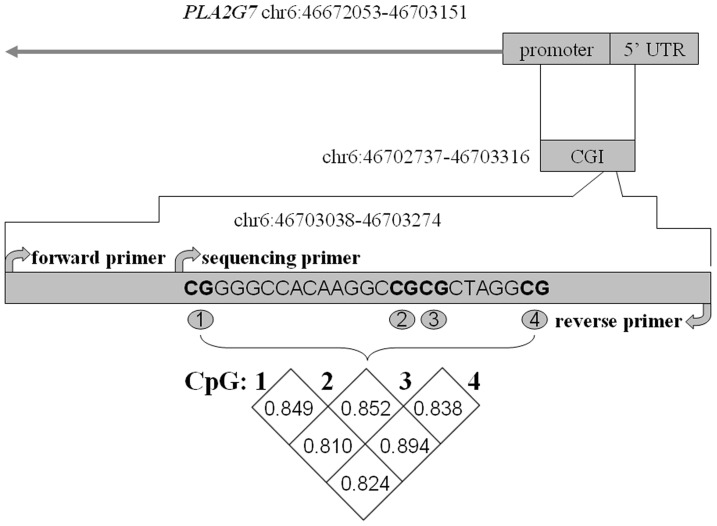
Significant correlation among the four CpGs in *PLA2G7* gene promoter.

As shown in Figure 1, the bisulphate pyrosequencing assay was carried on a fragment (hg19, chr6:46702737–46703316) in the promoter region of *PLA2G7* gene. This fragment contained 4 CG sites that could be measured to evaluate the methylation levels of *PLA2G7* gene promoter. Significant correlation of the DNA methylation levels was found among these 4 CpGs (Figure 1, r >0.8, P<0.0001). No gender difference was observed for the promoter DNA methylation levels of *PLA2G7* gene (Table 1, adjusted P = 0.226). There was a significant higher promoter DNA methylation of *PLA2G7* gene in the CHD cases than in the non-CHD controls (Table 2, adjusted P = 0.025).

**Table 1:** Characteristics of subjects according to gender^a^.

Characteristics	Men (n = 36)Mean±s.e.	Women (n = 36)Mean±s.e.	P value
Age	62.0±5.4	62.2±5.4	0.896
TG (mmol/L)	2.39±0.82	2.75±0.80	0.054
TC (mmol/L)	4.19±0.85	4.64±0.84	0.060
HDL (mmol/L)	1.08±0.20	1.20±0.25	0.173
LDL (mmol/L)	1.59±0.90	1.54±0.95	0.444
ApoA I (g/L)	0.88±0.14	0.91±0.13	0.789
ApoB (g/L)	0.63±0.15	0.67±0.16	0.226
ApoE (g/L)	4.2±1.2	6.8±8.3	0.005^d^
Lp(a) (g/L)	0.27±0.36	0.31±0.33	0.298^c^
hs-CRP (mg/L)	3.2±3.4	3.5±3.5^b^	0.664^c^
ALB (g/L)	42.2±4.1	41.4±3.2	0.126
GLB (g/L)	22.6±3.7	25.4±3.6	0.112
A/G	1.9±0.3	1.7±0.3	0.033
ALT (IU/L)	24±14	23±15	0.502
AST (IU/L)	21±7	22±8	0.808
ALP (IU/L)	60±15	70±18	0.130
GGT(IU/L)	29±20	25±25	0.929^c^
mean *PLA2G7* methylation (%)	5.08±2.65	5.15±3.29	0.226

a P values were adjusted for the history of smoking, diabetes and hypertension.

b An outlier due to the clerical error was eliminated.

c Log-transformation was used.

d Nonparametric rank test was applied.

**Table 2:** Characteristics of subjects from cases and controls^a^.

Characteristics	Cases (n = 36)Mean±s.e.	Controls (n = 36)Mean±s.e.	P value
Age	62.5±5.5	61.7±5.2	0.802
Gender (M/F)	18/18	18/18	1.000
TG (mmol/L)	2.66±0.86	2.48±0.80	0.554
TC (mmol/L)	4.49±0.90	4.34±0.86	0.591
HDL (mmol/L)	1.15±0.24	1.13±0.22	0.747
LDL (mmol/L)	1.51±0.98	1.62±0.86	0.700^c^
ApoA I (g/L)	0.90±0.11	0.90±0.16	0.845
ApoB (g/L)	0.66±0.17	0.63±0.15	0.686
ApoE (g/L)	6.5±8.4	4.5±1.2	0.910^d^
Lp (a) (g/L)	0.32±0.38	0.27±0.31	0.325^c^
hs-CRP (mg/L)	3.5±3.5	3.1±3.5^b^	0.257^d^
ALB (g/L)	41.6±3.8	42.0±3.6	0.487
GLB (g/L)	24.2±3.7	23.8±4.1	0.942
A/G	1.8±0.3	1.8±0.3	0.697
ALT (IU/L)	26±17	21±11	0.168
AST (IU/L)	23±9	21±6	0.398
ALP (IU/L)	65±19	65±16	0.912
GGT (IU/L)	28±26	27±20	0.721^c^
mean *PLA2G7* methylation (%)	6.41±2.62	4.98±3.06	0.025

a P values were adjusted for the history of smoking, diabetes and hypertension.

b An outlier due to the clerical error was eliminated.

c Log-transformation was used.

d Nonparametric rank test was applied.

**Figure 2:**
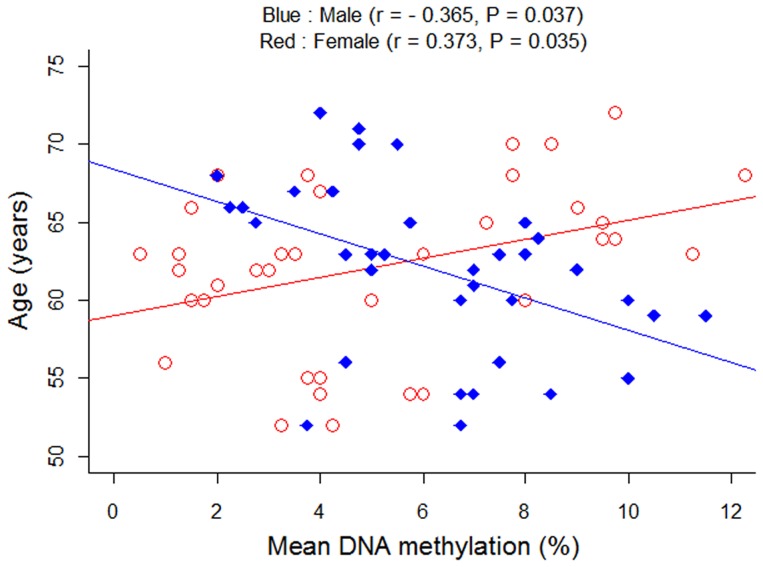
Correlation between *PLA2G7* methylation and age^a^. a) r values and P values were adjusted for the history of smoking, diabetes and hypertension.

**Figure 3:**
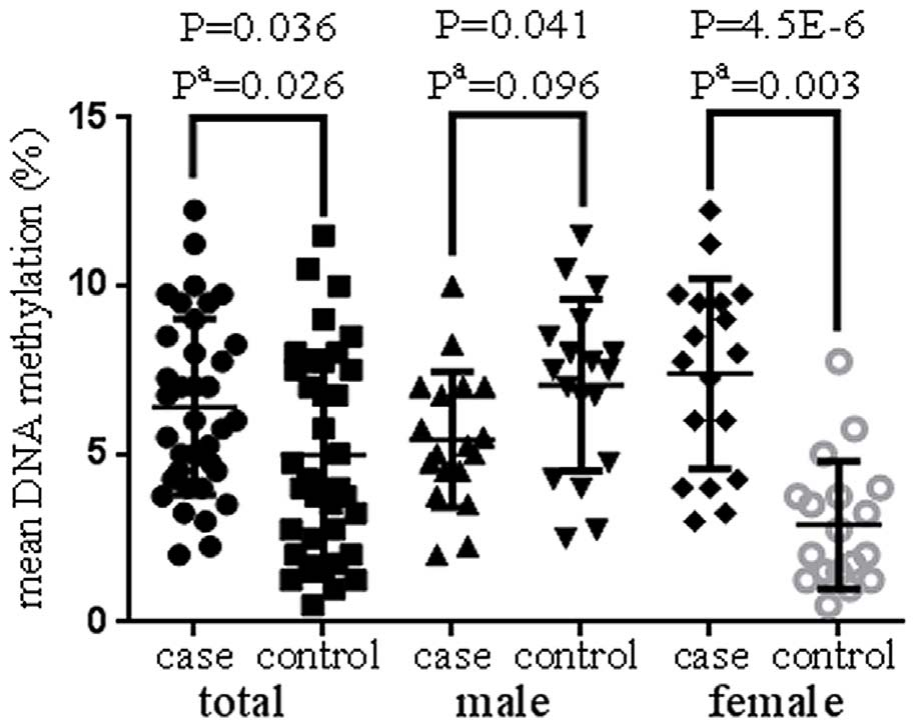
Comparison of *PLA2G7* methylation levels between cases and controls. a) P values were adjusted for age, the history of smoking, diabetes and hypertension.

It was intriguing that we observed a gender-specific pattern of the correlation between age and *PLA2G7* gene methylation (Figure 2, males: adjusted r = −0.365, adjusted P = 0.037; females: adjusted r = 0.373, adjusted P = 0.035). A further breakdown analysis by gender found that the significant association only existed in the female subgroup (Figure 3, females: uncorrected P = 4.5E−6, adjusted P = 0.003; males: uncorrected P = 0.041, adjusted P = 0.096). To note, the adjusted P values were corrected by age, the history of smoking, diabetes and hypertension. A further two-way ANOVA analysis also showed there was a significant interaction between gender and status of CHD for the methylation level of *PLA2G7* gene (gender*CHD: P = 6.04E−7).

**Figure 4:**
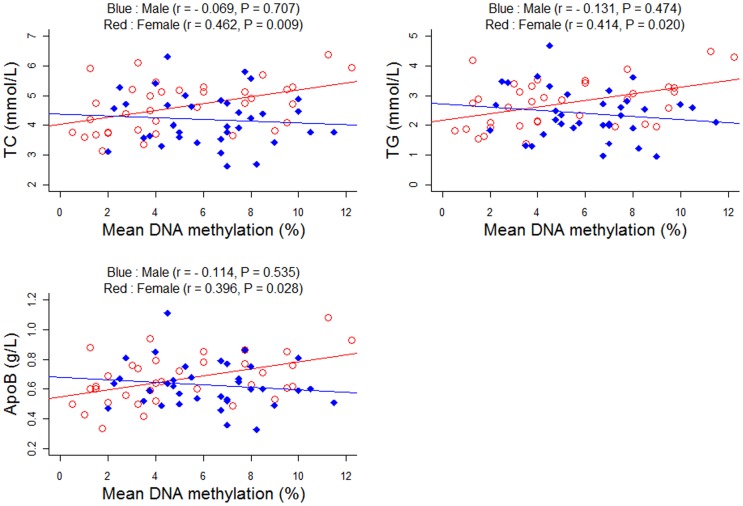
Pearson correlation between *PLA2G7* DNA methylation and TC, TG, ApoB^a^. a) r values and P values were adjusted for age, the history of smoking, diabetes and hypertension.

As shown in Table 1 and 2, a total of 16 phenotypes were involved in the present study. Among these phenotypes, only the ratio of albumin to globulin (A/G) showed a significant difference between men and women (Table 1, adjusted P = 0.033). No significant association was observed between A/G phenotype and CHD risk in either the female subgroup (P = 0.293) or the male subgroup (P = 0.716). And there is no correlation between the *PLA2G7* methylation and A/G phenotypes in either the female subgroup (plot 3b, r = −0.062, P = 0.739) or the male subgroup (r = −0.050, P = 0.786). No significant association of the rest phenotypes with the risk of CHD was found (Table 2). Interestingly, a significant female-specific association was found between *PLA2G7* promoter DNA methylation and phenotypes including TC, TG and ApoB (Figure 4, adjusted P<0.03 in females; adjusted P>0.4 in males).

**Figure 5:**
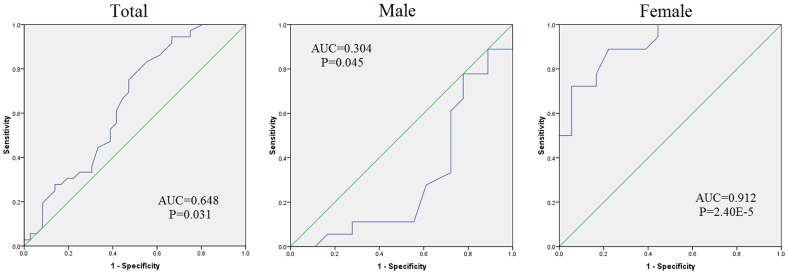
ROC curves of *PLA2G7* in total, male and female samples.

As shown in Figure 5, the receiver operating characteristic (ROC) analyses of curves showed that *PLA2G7* promoter methylation could predict the risk of CHD in the total samples (Figure 5, area under curve (AUC) = 0.648, P = 0.031). The subgrouped analyses showed a female-dependent effect of *PLA2G7* methylation in the prediction of CHD (Figure 5, males: AUC = 0.304, P = 0.045; females: AUC = 0.912, P = 2.40E−5).

## Discussion

CHD cases were observed to have significantly higher genomic DNA methylation in peripheral lymphocytes in comparison to non-CHD controls [39]. In addition, candidate epigenetics analysis showed that altered *ABCA1* promoter DNA methylation was associated with the risk of CHD [10]. In the present study, we had recruited 36 cases and 36 gender- and age-matched controls to test the association between DNA methylation of *PLA2G7* gene and the risk of CHD. We found that *PLA2G7* methylation changes with aging in a gender-specific pattern, which accords with the previous observation that the pattern of DNA methylation changes individually over time [40].

*PLA2G7* is the coding gene for Lp-PLA2 whose abnormal activity can cause high risk of CHD [30] and may serve as a diagnostic marker for CHD [27]. However, controversies remained in the association between *PLA2G7* variants and the risk of CHD [27], [30], [31], [41], [42]. The present study demonstrated that *PLA2G7* methylation changes with aging differently in two genders. Its female-specific contribution to the risk of CHD provides epigenetic clues to explain the inconsistency in the epidemiological studies.

Epidemiologic evidence from observational studies and randomized clinical trials identified high risk factors of CHD among older men and women [43]. Among the conventional risk factors, diabetes mellitus and hyperlipidemia was shown to impact more on women while smoking was found to affect more on men [44]. To test the difference between male cases and male controls, the unadjusted P value reached the significant level (P<0.05, Figure 3). However, the significance disappeared after corrected by age, the history of smoking, diabetes and hypertension (P = 0.096). So we speculate that environmental factors such as aging, smoking, diabetes and hypertension may play their roles in the risk of CHD through affecting *PLA2G7* methylation.

Gender difference exists in both the age of onset [45] and the long-term prognosis of CHD patients [46]. Estrogen regulates the enzymatic activity of PLA2G7 protein and thus causes significant changes in the metabolism of cholesterols and apolipoproteins [47]–[49]. In the present study, we observed a gender-dependent correlation between age and *PLA2G7* methylation, and an interaction between *PLA2G7* methylation and gender. TC, TG, and ApoB are known as important risk factors of CHD [50], [51]. And these findings suggest that *PLA2G7* methylation may exert its effects on the risk of CHD by regulating the levels of TC, TG, and ApoB in females.

*PLA2G7* methylation was shown to be controlled by the interactive effect of CHD status and gender. Female cases had a higher *PLA2G7* methylation level than female controls while it was the opposite in males, although the latter comparison was not significant after correction with confounding factors such as smoking and status of diabetes and hypertension. The trend of correlation between *PLA2G7* methylation and age was clearly opposite in the two genders. The ROC curves showed a much higher accuracy of *PLA2G7* methylation to predict CHD in females than in males.

Although there is a paucity of evidence showing a gender-dimorphism of methylation-mediated *PLAG7* expression in CHD, we observe a possible connection with *APOE* gene that may help explain our results. As shown in the Figure S1, there is an *APOE*-dependent correlation between *PLA2G7* expression and aging in mice (F = 12.42, P = 0.0074) [52]. Moreover, a female-specific interaction was found between *APOE4* genotype and the metabolism of HDL [53], which is an important protective factor in cardiovascular diseases including CHD. Another piece of evidence has shown a significant association between *APOE* expression and aging [54]. All the above evidence about *APOE* gene might give a plausible explanation for the sex-dimorphism of the association of *PLA2G7* promoter methylation with aging in CHD, although cautions need to be taken without direct supportive evidence. Future investigation of *APOE* interaction with *PLA2G7* is needed to confirm this speculation.

There were some limitations in our study. Firstly, the sample size in our study is relatively small. Future investigation with more samples needs to be performed to confirm our findings. Secondly, only a fragment of the CGI was selected to stand for the whole promoter of *PLA2G7*. Thirdly, *PLA2G7* methylation was measured in the whole peripheral blood which contained the DNA from a mixture of lymphocytes, granulocytes and other cell types. Fourthly, although we tried our best to control the confounding factors that may affect the methylation level of *PLA2G7*, there existed a possibility of an unknown factor that might confound the alteration of *PLA2G7* methylation. Fifth, we didn’t explore the mechanism why *PLA2G7* methylation correlated with TG, TC and ApoB in females. The exact interactions among them remained to be explained in the future work. Sixth, some P values in our study will not retain significance after being corrected by the number of tests. Therefore, we can’t exclude a chance of random positive findings among these results. Finally, the ages of female samples in our study range from 52 to 72. The mean age of natural menopause was 48.72±3.51 for Chinese female residents in city according to an epidemiological report in China [55]. As shown in Figure S2, the significance of association is mainly contributed by the eldly females. Thus, our results might be not feasible in the younger population.

In summary, we revealed *PLA2G7* methylation as a gender-dependent marker of aging and its female-specific association with the risk of CHD and biochemistry factors such as TC, TG, and ApoB. These findings could establish a molecular link between aging and the risk of CHD and thus contribute to a better understanding of the molecular mechanisms underlying the pathophysiology of CHD. The above clues may help improve the current clinical diagnosis and treatment of CHD.

## Supporting Information

Figure S1 **Comparison of** ***PLA2G7*** **methylation levels between cases and controls in different age groups in females^a^.** a) P values were adjusted for age, the history of smoking, diabetes and hypertension.
(TIF)

Figure S2 **Correlation between** ***PLA2G7*** **methylation and age^a^.**
(TIF)

Table S1 **Primer information of** ***PLA2G7*** **methylation assay.**
(DOC)
