# Supplementary figures and images for: Elevated PLA2G7 Gene Promoter Methylation as a Gender-Specific Marker of Aging Increases the Risk of Coronary Heart Disease in Females
Source: PLoS One. 2013 Mar 28;8(3):e59752. doi: 10.1371/journal.pone.0059752 (PMC3610900; doi:10.1371/journal.pone.0059752)

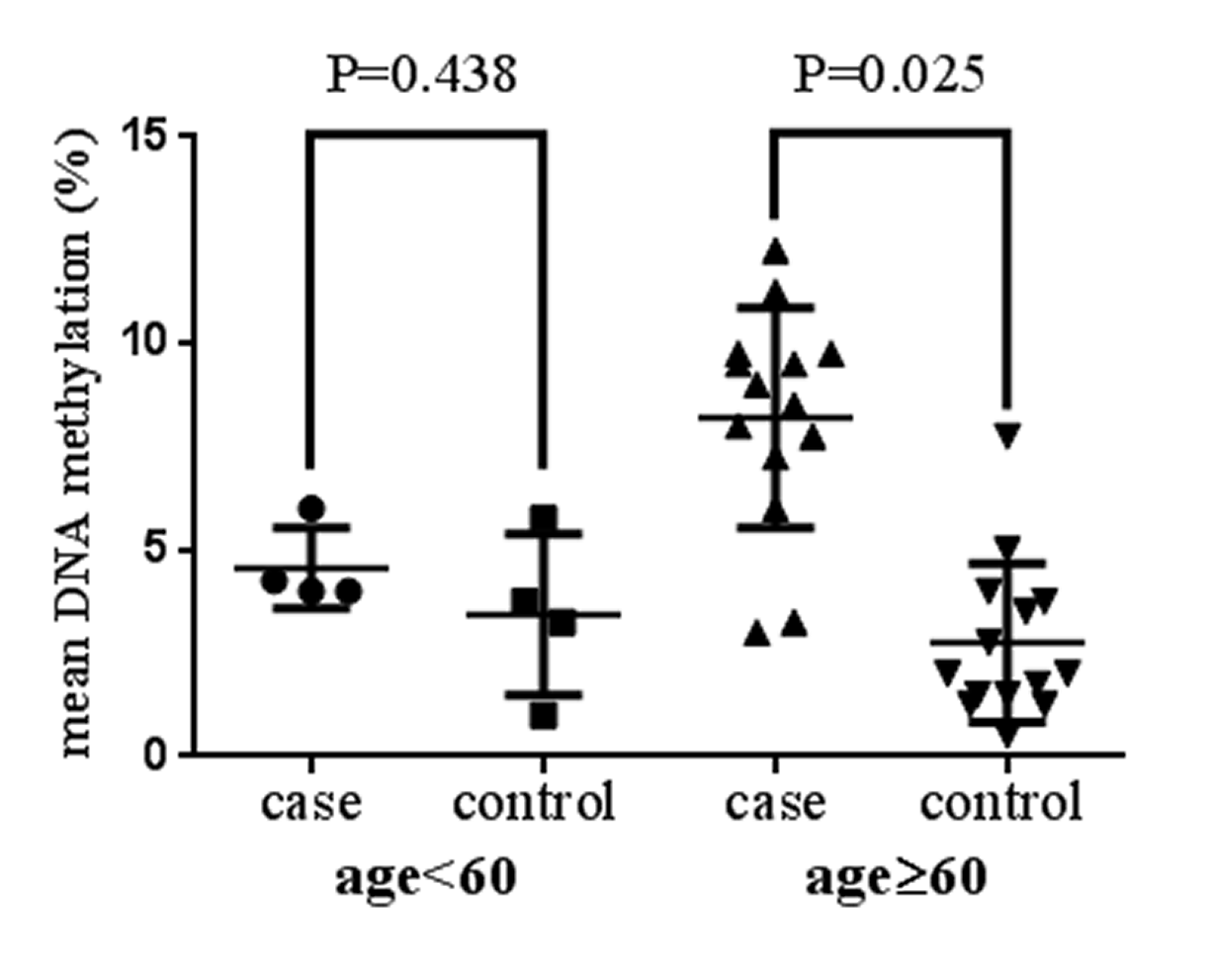

Supplement: Figure S1 — Comparison of PLA2G7 methylation levels between cases and controls in different age groups in femalesa. a) P values were adjusted for age, the history of smoking, diabetes and hypertension. (TIF) [file pone.0059752.s001.tif]

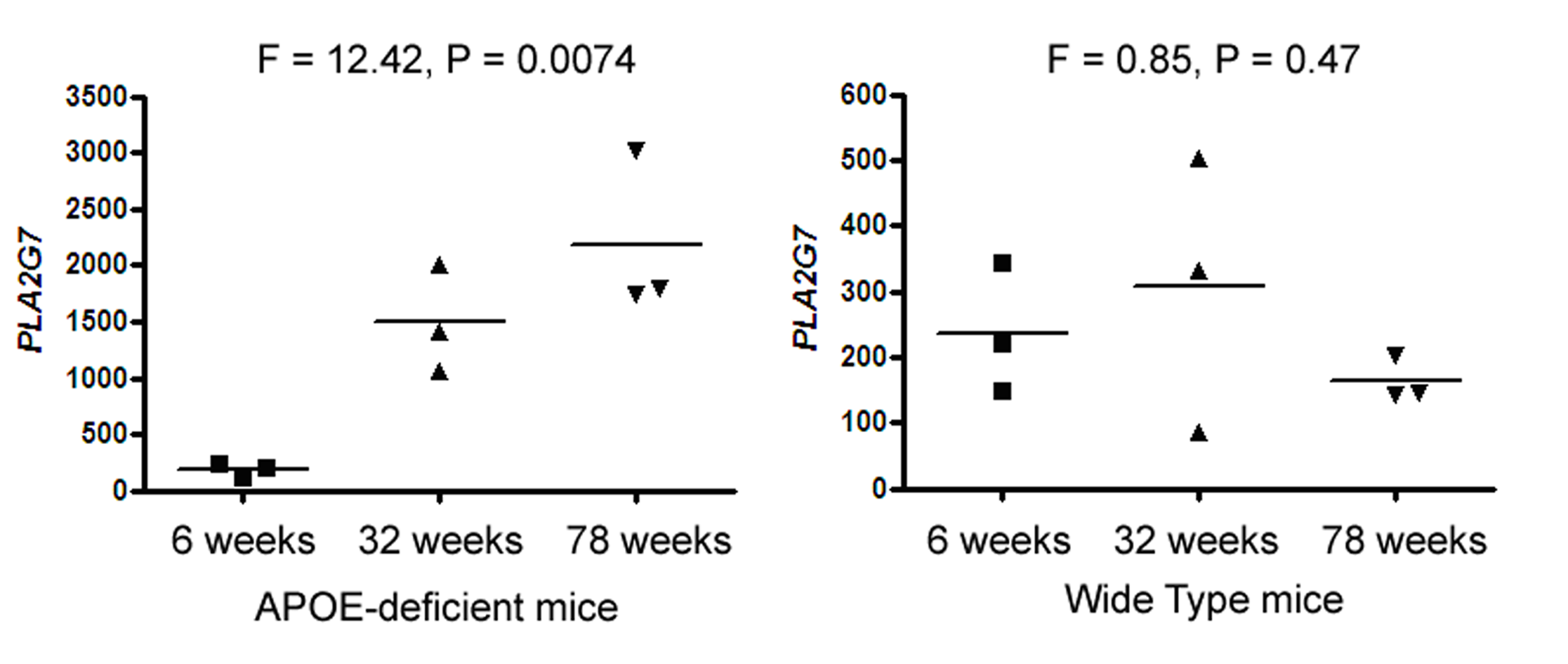

Supplement: Figure S2 — Correlation between PLA2G7 methylation and agea. (TIF) [file pone.0059752.s002.tif]
