# Supplementary material for: Elevated PLA2G7 Gene Promoter Methylation as a Gender-Specific Marker of Aging Increases the Risk of Coronary Heart Disease in Females
Source: PLoS One. 2013 Mar 28;8(3):e59752. doi: 10.1371/journal.pone.0059752 (PMC3610900; doi:10.1371/journal.pone.0059752)
Supplement: Table S1 — Primer information of PLA2G7 methylation assay. (DOC) [file pone.0059752.s003.doc]

Table S1: Primer information of *PLA2G7* methylation assay.

| Group | DNA Sequence |
| --- | --- |
| Forward primer | 5’-GTTTTGGGGAGGGTGTTG-3’ |
| Reverse primer | 5’-Biotin-ACCAACCCCTATCCCCCTAACTA-3’ |
| Sequencing primer | 5’-AGTATTATTAGGGGAGGA-3’ |
